# Antibiotic Use and Care-Seeking Practices for Childhood Diarrhea and Respiratory Illnesses in Community Settings in Bangladesh: A Cross-Sectional Caregiver Survey

**DOI:** 10.3390/antibiotics15060603

**Published:** 2026-06-13

**Authors:** Sampa Dash, Eva Sultana, Md. Razibur Rahman, Farina Naz, Mohammad Ali, Abu S. G. Faruque, Subhra Chakraborty

**Affiliations:** 1 Nutrition Research Division, International Centre for Diarrheal Disease Research, Bangladesh (icddr,b), Dhaka 1212, Bangladesh; sampa.dash1@icddrb.org (S.D.); eva.sultana1@icddrb.org (E.S.); razibur.rahman@icddrb.org (M.R.R.); farina.naz@icddrb.org (F.N.); md.ali@icddrb.org (M.A.); gfaruque@icddrb.org (A.S.G.F.); 2 Department of International Health, Johns Hopkins Bloomberg School of Public Health, Johns Hopkins University, Baltimore, MD 21218, USA; 3 Division of Infectious Diseases, School of Medicine, Johns Hopkins University, Baltimore, MD 21218, USA

**Keywords:** antibiotics, Bangladesh, care-seeking, diarrhea, respiratory illness

## Abstract

**Background:** Antimicrobial resistance, driven by inappropriate use and overuse of antibiotics, is a major public health threat. Diarrhea and respiratory illness are the leading causes of pediatric healthcare visits in low- and middle-income countries like Bangladesh. Despite clear WHO guidelines recommending limited use of antibiotics for these conditions, potentially inappropriate or non-prescription antibiotic use remains a concern. **Methods:** We interviewed caregivers of 3025 under-5 children via cellphones to assess common illnesses, associated care-seeking practices, and antibiotic use for diarrhea and respiratory illnesses experienced by their children in the prior 14 days. Caregivers were identified through hospital outpatient screening and were contacted over the phone for the interview at least two months after that hospital visit. **Results:** Among the participants, 116 (3.8%) reported diarrheal disease and 570 (18.8%) experienced respiratory illness during the preceding 2-week recall period. Among the children with diarrhea, 52.6% received antibiotics, and 73.8% obtained them over the counter from pharmacies. Among those with respiratory illness, 26.3% received antibiotics, and 58% procured them from local drugstores without a prescription from a registered physician. For diarrhea, azithromycin and metronidazole were the commonly used antibiotics, while for respiratory illness, cefixime and azithromycin were frequently used. Notably, 68% of the diarrheal children either sought care from local drugstores, were self-medicated, or did not receive any formal treatment. Conventional practice, long wait times at healthcare facilities, distance, and poverty were the main reasons for not seeking care from a registered healthcare provider. **Conclusions:** Understanding community-level antibiotic use and care-seeking behavior is essential to strengthening antibiotic stewardship and child health programs. Our findings suggest the need for context-sensitive community education, improved access to appropriate care, and enforcement of regulations restricting the over-the-counter sale of antibiotics to curb irrational and excessive antibiotic use.

## 1. Introduction

The inappropriate use and overuse of antibiotics for common pediatric illnesses remains a critical public health concern in low- and middle-income countries (LMICs) such as Bangladesh, where diarrheal diseases and respiratory illnesses, caused by either allergic reaction or infection by organisms, are the most common causes for health facility and pharmacy visits [1,2,3]. Antimicrobial resistance, including resistance to antibiotics, antivirals, antifungals, and antiparasitic agents, is a major global public health threat. In 2019 alone, globally, 1.27 million deaths occurred because of the rise in drug-resistant pathogens [4]. The misuse and overuse of antibiotics in humans, animals, and plants are the main drivers in the emergence of drug-resistant pathogens [4,5]. The children of LMICs are at higher risk of inappropriate antibiotic exposure [6]. The MAL-ED, a multi-country birth cohort study, reported that 44% of watery diarrhea episodes, 60% of episodes of fieldworker-confirmed acute lower respiratory tract illness, and 40% of episodes of upper respiratory illness among children aged below 5 years were treated with antibiotics [7]. Another multi-country prospective case–control study conducted in three Sub-Saharan African countries reported that 77.3% of the children were prescribed antibiotics without any indication [8]. According to a WHO report, in developing and transitional countries, only 40% of primary care patients in the public sector and 30% of those in the private sector are treated according to standard treatment guidelines with antimicrobials [9,10].

Diarrheal disease continues to be a major public health problem for under 5 children globally, particularly in LMICs. Despite a noteworthy decrease in diarrhea-related deaths in the last two decades, in 2024, the WHO reported that it is still the third leading cause of death in children under 5 years old globally, killing nearly 443,832 children aged 0–59 months yearly [11]. Bangladesh is one of the countries with the highest burden of pediatric diarrheal disease, driven by a high prevalence of enteric infections, inadequate water, sanitation, and hygiene (WASH) conditions, and widespread childhood undernutrition. WHO treatment guidelines specifically mentioned, based on the severity of the dehydration, oral rehydration solution (ORS) intake and/or infusion of intravenous (IV) electrolyte solution, along with zinc supplementation and continued feeding, are recommended treatments for diarrhea in children, reserving antibiotics only for cases with bloody diarrhea (probable shigellosis), suspected cholera, and for immunocompromised or severely malnourished patients [12]. Despite the guidelines, childhood diarrheal illness is one of the top reasons for the overuse of antibiotics in LMICs, though a high proportion of childhood diarrheal episodes are viral or self-limiting. A recent study conducted by our team in hospital settings among 8294 children reported that over 50% of children with diarrhea received antibiotics before being presented to the only tertiary care hospital in the locality, while only 6% had dysentery [13]. Inappropriate antibiotic use for diarrheal disease or other conditions not only increases economic burden on families and the health system but also drives the rise in antimicrobial resistance (AMR) in both diarrheal and commensal pathogens [14,15]. According to the National Antimicrobial Resistance Surveillance report for Bangladesh, 2016–2023, antibiotic resistance in Bangladesh has increased by 11% over the past 5 years [16]. A systematic review conducted on antibiotic resistance in Bangladesh, also reported a high prevalence of resistance across most pathogens [5]. Many of the common first-line drugs may become ineffective, as reported in antibiotic susceptibility testing [5].

Care-seeking behavior plays an important role in shaping treatment patterns for childhood illnesses. In LMICs, particularly in Bangladesh, antibiotics are frequently consumed for viral and self-limiting conditions, such as watery diarrhea, common colds, and uncomplicated upper respiratory tract infections. The WHO has clear clinical guidelines advising against routine use of antibiotics in these conditions [12,17]; however, antibiotics are frequently consumed without a prescription from a registered physician and in the absence of appropriate diagnostic confirmation. Caregivers in rural Bangladesh often rely on informal or unqualified providers, such as local drugstore vendors [13]. This practice increases the likelihood of inappropriate antibiotic dispensing, inadequate clinical assessment, and delayed referral for severe illness. Understanding these behaviors at the community level is essential to strengthening child health programs, optimizing antibiotic stewardship, and improving alignment with national guidelines.

However, there are very few community-based research studies conducted in Bangladesh that simultaneously quantify antibiotic use and examine care-seeking practices for common childhood illnesses. Most existing studies are facility-based and do not adequately capture the real-world practices at the household and community levels. To address these gaps, the current study conducted a cross-sectional survey among caregivers of 3025 children under 5 years old in Mirzapur Upazila, a rural region of Bangladesh, to examine the prevalence of antibiotic use for diarrheal illness and other common childhood illnesses in the community. We also studied the practiced care-seeking behavior among the general population regarding diarrhea and respiratory illness. This study provides community-level evidence using structured phone call interviews, capturing treatment practices beyond formal healthcare settings, including the high reliance on local drugstores and widespread antibiotic use even in uncomplicated diarrheal episodes. The study findings contribute critical insights for policymakers, clinicians, and public health planners working to reduce child morbidity and promote rational antibiotic use in Bangladesh.

## 2. Results

### 2.1. Participants’ Characteristics

We studied a total of 3025 children under 5 years old. Among them, 58.4% of the children were aged between 24 and 59 months. The sample included 1525 males (50.4%) and 1500 females (49.6%). Of the total number of study children, 801 (26.5%) reported being ill in the preceding 14-day period. A total of 116 (3.8%) children had diarrhea in the last 14 days (Table 1).

### 2.2. Reported Childhood Illnesses

The distribution of childhood illnesses and corresponding antibiotic use is presented in Table 2. Of the total 801 children who reported being sick in the preceding 14 days, respiratory illness (RI) without fever constituted the largest proportion of illness episodes (62.3%), yet 22.7% of these children received antibiotics. Diarrheal illness accounted for 14.5% of reported cases, and more than half of these children (52.6%) were treated with antibiotics, despite most diarrheal episodes being uncomplicated. Approximately 51.7% of the children who had RI associated with fever received antibiotics. Among the children with diarrhea, 11 children had both RI and diarrhea.

### 2.3. Comparison of Diarrheal Children by Antibiotic Receipt

Among the 116 diarrheal children, 61 children (52.6%) received antibiotics. The median age (IQR) for the children who received antibiotics for diarrheal illness was 22 months (15–31 months) vs. 19 months (14–30 months) for children who did not receive antibiotics. Children who received antibiotics had a shorter duration of diarrhea (3 days vs. 4 days; *p* = 0.004), and 31.2% resolved diarrhea on average within less than three days. Fever was noted in more diarrheal children who received antibiotics than those who did not (45.9%, 95% CI = 33.7–58.6% vs. 20.0%, 95% CI = 11.3–32.8; *p* = 0.004). More children in the antibiotic-receiving group also received zinc and ORS (62.3%, 95% CI = 49.4–73.7 vs. 41.8%, 95% CI = 29.4–55.3; *p* = 0.027) (Table 3).

### 2.4. Barriers to Seeking Care at a Healthcare Facility for Childhood Diarrhea

Among the children with diarrhea, 79 (68%) received treatment from a local drugstore or self-medicated, rather than visiting a registered physician. When the reason behind that was asked, 46.8% of them said they usually seek treatment at the local drugstore. For 16.5% of the children, the reason was long waiting times in a health care facility, and for 17.7% of children, the long distance and related transport cost were the reasons for not seeking care at a health care facility (Table 4).

### 2.5. Antibiotic Usage Patterns for Childhood Diarrhea

**Figure 1:**
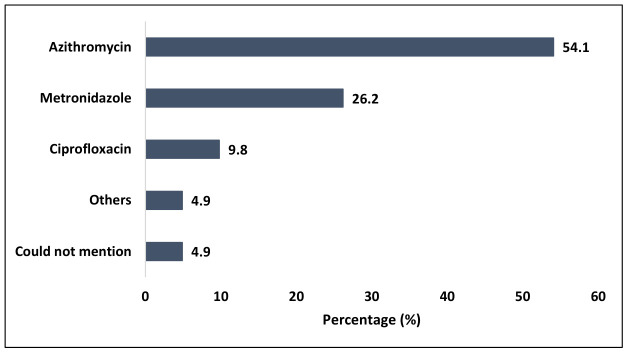
Types of antibiotics used for diarrheal children.

Among the 61 children who received antibiotics for diarrhea, 54.1% received azithromycin, and 26.2% received metronidazole. (Figure 1).

Among the 61 diarrheal children who received antibiotics, 45 (73.8%) obtained them from local drugstores, including licensed pharmacies and informal medicine shops. Among them, four children first sought care at the government health facility and were not given antibiotics. Then, they visited a local drugstore and received the antibiotic. One child was first treated at a local drugstore and not given antibiotics, but subsequently visited a private care facility and got antibiotics. For another child, the caregiver directly purchased antibiotics from a local drugstore without consulting a healthcare provider, as the caregiver already knew the name of the antibiotic used for treating diarrhea (Table 5).

### 2.6. Characteristics of Children with Respiratory Illness by Antibiotic Receipt

Table 6 describes the characteristics of children who experienced respiratory illness (RI) in the preceding 14 days, stratified by antibiotic use. A total of 570 (18.8%) children experienced RI, and about 26.3% of them received antibiotics. Most of the RI cases were uncomplicated, as they were not associated with any other symptoms. Children with RI who had accompanying fever were substantially more likely to receive antibiotics compared to those without fever (22.0% vs. 6.9%, *p* < 0.001). Similarly, the presence of diarrhea alongside RI was more common among children who received antibiotics than among those who did not (4.0% vs. 1.2%, *p* = 0.032).

### 2.7. Antibiotic Usage Pattern for Respiratory Illness

**Figure 2:**
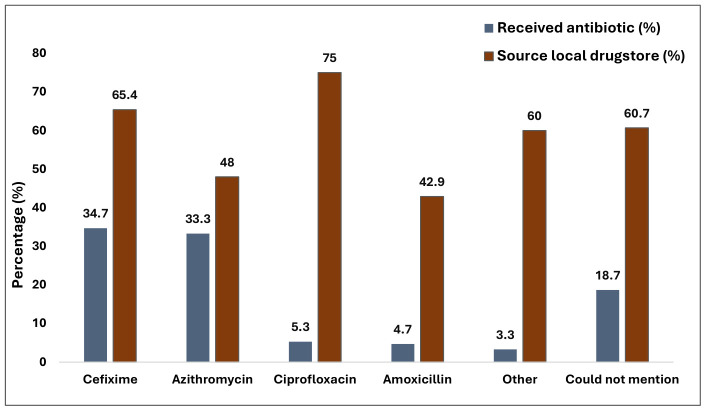
Types of antibiotics used for RI and the proportion of them received from local drugstores.

Among the children with RI who received antibiotics, 87 (58%, 95% CI: 49.87–65.72) children received the antibiotic from local drugstores over the counter. Around one-third of the antibiotic-receiving children with RI received cefixime, and about another one-third received azithromycin. About 65.38% of cefixime and 48% of azithromycin were obtained from local drugstores. Caregivers of 19.15% of children could not name the antibiotic. The rest received antibiotics like amoxicillin, ceftriaxone, cefpodoxime, ciprofloxacin, and metronidazole (Figure 2).

## 3. Discussion

In this community-based cross-sectional study, we observed substantial antibiotic use among young children for common childhood illnesses, particularly for diarrheal and respiratory illnesses. Most of the antibiotics were received from local drugstores.

In our study, one-fourth of the study participants (801) reported at least one episode of common childhood illness in the preceding 14 days of the interview. The most common reported illnesses were respiratory tract infection, diarrhea, and fever with nonspecific symptoms. Interestingly, all the sick children received some form of medication, such as antibiotics, antihistamines, or vitamin supplements. About 30.7% of the sick children received antibiotics, and 147 (59.8%) received them from a local drugstore without presenting any formal prescription from a registered physician.

Diarrhea is one of the major contributors to the consumption of antibiotics. Among the 116 children with diarrhea, 61 (52.6%) received antibiotics, despite clear WHO guidelines against the routine use of antibiotics for watery diarrhea, except in cases of suspected cholera; in our study, only 7.8% of the diarrheal children had dysentery. This finding is consistent with our earlier study among children with diarrhea at a tertiary care hospital in Mirzapur, which reported that 55% of the children received antibiotics before coming to the hospital, while only 6% had dysentery [13]. The present study extends this evidence to the community level, highlighting that inappropriate antibiotic use is also prevalent for mild diarrheal episodes that do not require hospital visits. The high prevalence of antibiotic use among children with diarrhea is consistent with previous evidence from Bangladesh and other LMICs, where antibiotics are frequently used for self-limiting diarrheal illnesses [8,13,18].

Overuse of antibiotics was also observed among children with respiratory symptoms, though the proportion was substantially lower than among children with diarrhea. About 26% of the children with RI received antibiotics, though those are mostly for the common cold, which might be due to seasonal allergies or viral causes. It is also notable that children who had a fever with RI (62 of 570 children with RI, 10.9%) are more likely to receive antibiotics (33, 53.2%). These results also support the findings from a secondary analysis of BDHS-14 data, which reported 39% of the children with ARI received antibiotics [19]. WHO guidelines do not recommend antibiotic use for uncomplicated respiratory tract infections [12,17,20]. These findings highlight a persistent and concerning gap between recommended case management of childhood illness and real-world treatment practices in the community.

Care-seeking behavior seems to play a crucial role in antibiotic consumption. Bangladesh has made remarkable progress in pediatric healthcare over the past few decades, which is reflected in the substantial reduction in under-five mortality and morbidity through an exceptionally high immunization coverage, enhanced maternal and child healthcare services and better management of common childhood illnesses. National programs like Expanded Programme on Immunization (EPI), and Integrated Management of Childhood Illness (IMCI) played a vital role in improving child health outcomes. Despite these achievements, challenges remain, particularly in rural and underserved areas where access to specialized pediatric care, diagnostic facilities, and trained healthcare professionals is limited. Consequently, local drugstores become the first point of contact for seeking care for pediatric illness.

About 60% (69 of 116 diarrheal children) of the caregivers of diarrheal children sought treatment at the local drugstores, and another 9% (10 of 116 diarrheal children) self-medicated rather than consulting registered physicians. Nearly 60% (41 of 69 children) of the children who sought care at local drugstores for diarrheal illness received antibiotics. The usual habit of seeking care at a local drugstore, long wait times at healthcare facilities, and distance to the health facilities were the most reported reasons for not receiving care from formal healthcare facilities. The same pattern was noted for the children who had RI. Of the 150 children who received antibiotics for RI, 58% obtained them from local drugstores. Although according to the Drugs and Cosmetics Act, 2023, antibiotics should only be dispensed with a prescription from a registered clinician, regulatory enforcement is often weak, allowing community pharmacies and drug sellers to dispense antibiotics without a prescription [21,22,23]. A study using the BDHS-22 also found that most children received antibiotics from a local drugstore without a prescription from a qualified physician [19]. These findings underscore the influential role of retail drugstores in managing pediatric illnesses in rural Bangladesh. Engaging this sector by educating vendors about the consequences of inappropriate antibiotic use and the proper implementation of law and legislation, by prohibiting sales of over-the-counter antibiotics, may play a vital role in improving antibiotic stewardship.

Notably, in this study, azithromycin was the most used antibiotic for diarrheal illness (54.1%) and the second most used antibiotic for RI (33.3%). Almost the same proportion of the children with RI used cefixime (34.7%) to treat RI in this study, and, interestingly, 65.4% of the cefixime was purchased directly from a local drugstore without consulting any physician. A cross-sectional study conducted in Bangladesh by interviewing 287 pharmacy staff about their knowledge and practice on dispensing non-prescribed antibiotics revealed that azithromycin is the most dispensed (76.7%) non-prescribed antibiotic [24]. The predominant use of such broad-spectrum antibiotics is concerning, especially when they can be bought over the counter without a prescription, given that those antibiotics are in the watch group of the WHO AWaRE classification, and used to treat some serious childhood illnesses like pneumonia, bronchitis, acute gastroenteritis, etc. [25]. The AMR surveillance report of Bangladesh reported that the effectiveness of several commonly used antibiotics decreased by up to 82% in 2023, compared to 71% in 2016 [16]. Frequent use of antibiotics for suspected infections without microbiological confirmation can cause antimicrobial resistance, and children may suffer from the detrimental effects of injudicious overuse of antibiotics. The widespread use of azithromycin—often prescribed empirically for presumed enteric infection—further reflects diagnostic uncertainty and non-guideline-based prescribing practices. Such patterns may accelerate the development of antimicrobial resistance among enteric pathogens and compromise the effectiveness of commonly used antibiotics.

In this study, about 95% of children received ORS for treatment of diarrhea, and 53% of children received zinc and ORS. Interestingly, children who received antibiotics are more likely to receive zinc (62.3% vs. 43.6%; *p* = 0.044). Zinc helps reduce diarrheal duration as reported by multiple studies [26,27]. In this study, children who received antibiotics had a shorter duration of diarrhea. However, given the cross-sectional and observational nature of the study, no causal relationship between antibiotic use and clinical recovery can be inferred. The observed association may be due to differences in healthcare-seeking behavior, caregivers’ level of education, socioeconomic status, illness severity, or underlying disease etiology, which could not be captured in this study.

## 4. Method

### 4.1. Study Design and Study Site

A cross-sectional survey of caregivers about their care-seeking practices at the community level was conducted among children under five years old in the Mirzapur Upazila (sub-district) for 7 months from July 2023 to January 2024. The caregivers were identified through hospital outpatient screening and interviewed by phone at least 2 months after that hospital visit. Mirzapur Upazila comprises 15 unions, located about 54 km northwest of Dhaka, the capital city of Bangladesh. This is home to approximately 474,658 people, including nearly 43,000 children under 5.

As part of an ongoing prospective case–control study titled “The Impact of Non-dysentery *Shigella* Infection on the Growth and Health of Children Over Time (INSIGHT)” [28], screening and data collection were conducted among children with diarrhea under 5 years of age at the pediatric outpatient department of Kumudini Women’s Medical College and Hospital (KWMCH), Mirzapur. Children presenting to the outpatient department with any illness or for vaccination, between 8:00 am and 5:00 pm were screened daily by trained study staff. Children who met the eligibility criteria for the INSIGHT study were screened and enrolled in accordance with the study protocol. In parallel, during routine screening, caregivers of all children visiting the outpatient department of KWMCH were approached, and their mobile phone numbers were collected after obtaining verbal consent. Caregivers were informed that they might be contacted later for a brief telephone interview related to child health.

During the first two months of the current study, approximately 5000 phone numbers of caregivers of under-5 children were collected from Kumudini Hospital. After a minimum interval of two months from that hospital visit, the study staff contacted caregivers by telephone to seek verbal consent for participation in a structured phone call interview. Interviews were conducted immediately when caregivers were available; if not, interviews were scheduled at a time convenient for the caregiver. Of the caregivers contacted, complete interview data were successfully collected from 3025 children using a structured questionnaire. The remaining caregivers could not be included due to unreachable phone numbers, non-response after repeated call attempts, or refusal to participate in the interview.

### 4.2. Sample Size

The sample size was calculated using the single population proportion formula, assuming a two-week prevalence of diarrhea of 4.7% and respiratory illness of 35.8% among children under 5 years of age in Bangladesh [29]. A 95% confidence level was used, with a precision of 2% for diarrhea and 3% for respiratory illness. The initial sample sizes were estimated to be 430 and 981 participants for diarrhea and respiratory illness, respectively, after rounding up. After applying a design effect of 2 and adjusting for an anticipated 10% non-response rate, the required sample sizes were 962 for diarrhea and 2180 for respiratory illness. Since the larger sample size was required for estimating respiratory illness prevalence with 3% precision, a minimum sample size of 2180 participants were considered necessary for the study. Ultimately, a total of 3025 caregivers of children under five years of age were interviewed, substantially exceeding the minimum required sample size (Supplementary File S1).

### 4.3. Data Collection and Analysis

Study staff interviewed the caregiver of each child once using a structured, field-tested questionnaire over the phone. Study staff called the caregiver of the study participant on weekdays between 9 am and 5 pm. They first introduced themselves to the caregivers and asked for their availability and permission for the interview. After obtaining verbal consent, the interview process was started. Primarily, the data were collected on paper and subsequently transferred to the Microsoft Excel database daily. These electronic records were routinely cross-checked against the paper documents to ensure accuracy and completeness. Data were cleaned and validated by the field supervisor twice a week, and any discrepancies identified were reviewed and corrected as required.

### 4.4. Variables

The outcome variable was antibiotic use, and age, sex, breastfeeding practices, care-seeking practices, and the medications used were predictor variables.

The caregivers were asked about any illness in the past 14 days before the interview. Diarrhea was confirmed by asking if that child passed ≥3 abnormally loose or watery stools in any 24 h period. The reported illness is considered respiratory illness when the symptoms involved the respiratory system, including both upper and lower respiratory tract (nose, throat, airways, lungs and other related structures). Questions were asked to determine care-seeking practices, where they seek treatment for diarrheal and respiratory illnesses, and other diseases, antibiotic use and its source, factors influencing care-seeking practices, breastfeeding practices, etc.

The question on antibiotic usage at home was asked to the caregiver to determine whether the child was given any antibiotic for that episode of diarrhea or other illness. To confirm that the reported drug is an antibiotic, the caregivers were asked if they had any physical proof, like the prescription or the bottle, strip or cover of the medicine, or description of the medicine (color, liquid or pills, dosage, price, formulation, whether mixing of any contents of the bottle with water was advised by the provider, etc.). To determine dysentery cases, caregivers were asked if the child passed any loose stool with blood. Questions regarding breastfeeding practices included the history of exclusive and mixed breastfeeding for the first 6 months, and current breastfeeding practices.

The common care-seeking practices for diarrhea were determined by the treatment used for diarrhea, where they sought care for diarrhea, source of antibiotics, and the use of ORS, zinc, antibiotics, probiotics, and folic acids. The other covariates were age, sex, onset date and time of diarrhea, the reason for not taking ORS at home, address, and demographic profile.

### 4.5. Statistical Analysis

The data were analyzed using STATA version 17.0 (Stata Corp, College Station, TX, USA). Frequencies and proportions were used to describe the characteristics of the study population. Continuous data (age of the children and duration of diarrhea) were presented as median and IQR as the data were not normally distributed; categorical data were represented as frequency and percentages. Associations between categorical variables were assessed using the Chi-square test. Fisher’s exact test was used when any expected cell count was less than 5. We conducted a non-parametric Mann–Whitney test to assess the association between continuous variables. The statistical significance of the analysis was determined when *p* < 0.05.

## 5. Strength and Limitation

This study has several strengths, including its large sample size and the collection of detailed information on antibiotic use for diarrheal and respiratory illnesses. However, some limitations should be acknowledged. While the contact numbers and initial consent of the caregivers were collected from the hospital, we interviewed the caregivers at least a 2-month after the index hospital visit. This time interval allowed this study to capture routine care-seeking behavior and antibiotic-use patterns at the community level beyond the influence of the hospital visit. Illness episodes and treatments were self-reported and therefore subject to recall bias, although this bias is likely minimal within 14 days. The relatively small number of diarrheal cases limits our ability to perform multivariable analyses. Additionally, as detailed medication data were collected only for diarrheal illnesses and respiratory illnesses, comparisons with other illnesses should be interpreted with caution. Future studies should explore preventive child health interventions, including vaccination coverage, WASH practices, and practices of other preventive measures, to better understand how reducing infectious disease burden may indirectly decrease community-level antibiotic consumption and support antimicrobial stewardship [30].

## 6. Conclusions

In conclusion, our findings suggest that inappropriate antibiotic use for childhood diarrheal illness remains widespread in rural Bangladesh and is strongly influenced by community care-seeking behavior and reliance on local drugstores. Interventions aimed at reducing unnecessary antibiotic exposure in children should prioritize caregiver education, strengthen regulation and engagement of retail pharmacies, and reinforce guideline-based management of childhood diarrhea. Addressing non-prescription antibiotic access, caregiver care-seeking barriers, and reliance on informal treatment sources is essential to curb the growing threat of antimicrobial resistance and may support improved, safer pediatric care in Bangladesh.

## Figures and Tables

**Table 1:** Characteristics of the study children.

Total Study Participants (*N* = 3025)	*n* (% of Total)	95% CI
Age of the children (months)		
1–5	49 (1.6)	1.2–2.1
6–11	217 (7.2)	6.3–8.2
12–23	991 (32.8)	31.1–34.5
24–59	1768 (58.4)	56.7–60.2
Sex		
Male	1525 (50.4)	48.7–52.2
Female	1500 (49.6)	47.8–51.4
Morbidity		
Participants suffered from at least one episode of illness in the last 14 days	801 (26.5)	24.9–28.1
Diarrhea in the last 14 days	116 (3.8)	3.2–4.6
Respiratory illness (RI) in the last 14 days	570 (18.8)	17.5–20.3
Family member experienced diarrhea	75 (2.5)	2.0–3.1
Breastfeeding practices		
Breastfed for 1st 6 months	2840 (93.9)	92.9–94.7
Exclusively breastfed for the 1st 6 months	369 (12.2)	11.1–13.4
Antibiotic received in the last 14 days	246 (8.1)	7.0–8.9

**Table 2:** Reported illnesses and antibiotics used among the children.

Name of the Illness (*n* = 801)	Total Patient *n* (%)		Antibiotic Received *n* (%)	
RI without fever	499	(62.3)	113	(22.7)
RI with fever	58	(7.2)	30	(51.7)
RI with skin rash	2	(0.3)	1	(50)
Diarrhea	116	(14.5)	61	(52.6)
Fever	82	(10.2)	23	(28.1)
Fever with abdominal pain	1	(0.1)	1	(100)
Skin rash	34	(4.2)	10	(29.4)
Other	9	(1.1)	5	(55.6)

**Table 3:** Comparison of the children who received antibiotics and those who did not for diarrheal illness.

	Without Antibiotic		Antibiotic Received		*p*-Value
	*n* = 55		*n* = 61		
	*n* (%)	95% CI	*n* (%)	95% CI	
Age of the children (months)					
Median (IQR)	19 (14–30)		22 (15–31)		0.815 ^1^
1–5	3 (5.5)	1.7–15.8	2 (3.3)	0.8–12.4	
6–11	8 (14.6)	7.4–26.7	7 (11.5)	5.5–22.4	
12–23	22 (40.0)	27.8–53.6	29 (47.5)	35.2–60.2	
24–59	22 (40.0)	27.8–53.6	23 (37.7)	26.4–50.6	
Sex					
Male	25 (45.5)	32.7–58.8	34 (55.7)	43.0–67.8	0.273 ^2^
Breastfeeding practices					
Currently breastfeeding	40 (72.7)	59.4–83.00	37 (60.7)	47.8–72.2	0.173 ^2^
Breastfed for the first 6 months	53 (96.4)	86.3–99.1	56 (91.8)	81.6–96.6	0.307 ^2^
Exclusively breastfed for the first 6 months	6 (10.9)	4.9–22.5	6 (9.8)	4.4–20.4	0.851 ^2^
Clinical characteristics					
Dysentery	4 (6.9)	3.6–19.3	5 (8.6)	4.1–14.4	1.000 ^3^
Duration of diarrhea					
Median (IQR)	4 (3–6)		3 (2–4)		0.004 ^1^
1–2 days	5 (9.1)	3.8–20.3	19 (31.2)	20.7–44.00	
3–5 days	35 (63.6)	50.1–75.4	35 (57.4)	44.6–69.3	
6–7 days	12 (21.8)	12.7–34.8	3 (4.9)	1.6–14.4	
8–10 days	3 (5.5)	1.7–15.8	2 (3.3)	0.8–12.4	
11–14 days	0	0.89–13.66	2 (3.3)	0.8–12.4	
Associated other morbidity					
Fever	11 (20)	11.3–32.8	28 (45.9)	33.7–58.6	0.004 ^2^
Abdominal Pain	12 (21.8)	12.7–34.8	8 (13.1)	6.6–24.3	0.220 ^2^
Vomiting	24 (43.6)	31.1–57.1	33 (54.1)	41.4–66.3	0.265 ^2^
Common cold	20 (36.4)	24.6–50.0	23 (37.7)	26.4–50.6	0.882 ^2^
Medications received					
ORS	50 (90.9)	79.7–96.2.	60 (98.4)	89.0–99.8	0.070 ^2^
Zinc	24 (43.6)	31.1–57.1	38 (62.3)	49.4–73.7	0.044 ^2^
Zinc and ORS	23 (41.8)	29.4–55.3	38 (62.3)	49.4–73.7	0.027 ^2^
Probiotic	11 (20.0)	11.3–32.8	11 (18.1)	10.2–29.9	0.787 ^2^
Folic Acid	4 (7.3)	2.7–18.1	5 (8.2)	3.4–18.4	1.000 ^3^
Care-seeking practices for diarrheal illness					
None/self-medication	9 (16.4)	6.7–38.3	1 (1.6)	0.2–11.0	0.006 ^3^
Local drugstore	28 (50.9)	37.8–64.0	41 (67.2)	54.4–77.9	0.074 ^2^
Private facility	8 (14.6)	7.4–26.7	7 (11.5)	5.5–22.4	0.623 ^2^
Government primary healthcare facility	3 (5.5)	1.7–15.8	2 (3.3)	0.8–12.4	0.667 ^3^
Government secondary and tertiary-level healthcare facility	7 (12.7)	6.1–24.6	10 (16.4)	9.0–28.1	0.577 ^2^

^1^ Non-parametric Mann–Whitney test was used to determine significance; ^2^ Chi-square test was used to determine significance; ^3^ Fisher’s exact test was used to assess statistical significance.

**Table 4:** Reason for not seeking care at a health facility for diarrheal illness.

*N* = 79	*n* (%)	95% CI
Long waiting time at the health facilities	13 (16.5)	9.7–26.6
Long distance and transportation cost	14 (17.7)	9.9–35.4
Usually seek care from the local drugstore	37 (46.8)	35.9–58.1
Financial problem	11 (13.9)	7.8–23.7
Others	4 (5.1)	1.9–13.00

**Table 5:** Sources of antibiotics received for diarrheal illness.

Name of the Sources of Antibiotics	*N* (%)	95% CI
Local drugstore	45 (73.8)	61.0–83.5
Private healthcare facility	8 (13.2)	5.2–33.2
Govt. primary-level healthcare facility	7 (11.5)	4.6–31.9
Self	1 (1.6)	0.2–11.3

**Table 6:** Characteristics of those who experienced RI in the last 14 days.

Total Children with RI = 570	Without Antibiotic		Antibiotic Received		*p*-Value
	*n* = 420		*n* = 150		
	*n* (%)	95% CI	*n* (%)	95% CI	
Age of the children (months)					
Median (IQR)	24 (16–37)		21 (13–35)		0.116 ^1^
1–5	6 (1.4)	0.6–3.2	3 (2.0)	0.6–6.0	
6–11	42 (10.0)	7.5–13.3	24 (16.0)	11.0–22.8	
12–23	147 (35.0)	30.6–39.7	57 (38.0)	30.6–46.0	
24–59	225 (53.6)	48.8–58.3	66 (44.0)	36.3–52.1	
Sex					
Male	226 (53.8)	49.0–58.5	83 (55.3)	47.3–63.1	0.748 ^2^
Female	194 (46.2)	41.5–51.0	67 (44.7)	36.9–52.7	0.748 ^2^
Breastfeeding practices					
Currently breastfeeding	243 (57.9)	53.1–62.5	88 (58.7)	50.6–66.3	0.863 ^2^
Breastfed for the first 6 months	390 (92.9)	85.0–94.8	142 (94.7)	89.7–97.3	0.446 ^2^
Exclusively breastfed for the first 6 months	42 (9.8)	7.3–13	16 (10.7)	6.6–16.7	0.751 ^2^
Associated other morbidity					
Fever	29 (6.9)	4.9–9.8	33 (22.0)	16.1–29.4	<0.001 ^2^
Diarrhea	5 (1.2)	0.5–2.8	6 (4.0)	1.8–8.6	0.032 ^2^

^1^ Non-parametric Mann–Whitney test was used to determine significance; ^2^ Chi-square test was used to determine significance.

## Data Availability

All data generated or analyzed during this study are included in this published article. Any additional data related to this paper are available upon request to the corresponding author, email: schakr11@jhu.edu.

## Supplementary Materials

The following supporting information can be downloaded at: https://www.mdpi.com/article/10.3390/antibiotics15060603/s1, File S1: Sample Size Calculation; File S2: Common childhood illness and care-seeking practices for childhood Diarrhea and respiratory illnesses in the community in Bangladesh.
