# Supplementary material for: Antibiotic Use and Care-Seeking Practices for Childhood Diarrhea and Respiratory Illnesses in Community Settings in Bangladesh: A Cross-Sectional Caregiver Survey
Source: Antibiotics (Basel). 2026 Jun 13;15(6):603. doi: 10.3390/antibiotics15060603 (PMC13296247; doi:10.3390/antibiotics15060603)
Supplement: Supplementary file 1 [file antibiotics-15-00603-s001.zip › antibiotics-4326903-supplementary-file S1.pdf]

Supplementary file S1:
Sample Size Calculation:

The required formula for sample size calculation for estimating a single proportion is:

$$n = \frac{Z_{1-\alpha/2}^{2} P(1 - P)}{d^2}$$

Where,

- n= required sample size
- Z = z-score corresponding to the desired confidence level
- P = expected prevalence of the parameter
- d = margin of error (desired precision)

**Indicators**	**Diarrhea**	**Respiratory illness**
Expected prevalence (P)	4.7%	35.8%
Level of significance (α)	5%	5%
Z-score corresponding	1.96	1.96
Margine of error/Desired Precision (d)	2%	5%
Design Effect (DEFF)	2	3
Base sample size rounded up (before DEFF)	430	981
Sample size after applying DEFF (n × 2)	865	1962
Attrition (%)	10%	10%
Required minimum sample size (n)	**962**	**2180**

The sample size was calculated using the single population proportion formula, assuming a two-week prevalence of diarrhea of 4.7% and respiratory illness of 35.8% among children under five years of age in Bangladesh [29]. A 95% confidence level was used, with a precision of 2% for diarrhea and 3% for respiratory illness. The initial sample sizes were estimated to be 430 and 981 participants for diarrhea and respiratory illness, respectively, after rounding up. After applying a design effect of 2.0 and adjusting for an anticipated 10% non-response rate, the required sample sizes were 962 for diarrhea and 2180 for respiratory illness. Since the larger sample size was required for estimating respiratory illness prevalence with 3% precision, a minimum sample size of 2180 participants was considered necessary for the study. Ultimately, a total of 3,025 caregivers of children under five years of age were interviewed, substantially exceeding the minimum required sample size.
