# Supplementary material for: Antibiotic Use and Care-Seeking Practices for Childhood Diarrhea and Respiratory Illnesses in Community Settings in Bangladesh: A Cross-Sectional Caregiver Survey
Source: Antibiotics (Basel). 2026 Jun 13;15(6):603. doi: 10.3390/antibiotics15060603 (PMC13296247; doi:10.3390/antibiotics15060603)
Supplement: Supplementary file 1 [file antibiotics-15-00603-s001.zip › antibiotics-4326903-supplementary-file S2.pdf]

Supplementary file S2

Common childhood illness and care-seeking practices for childhood Diarrhea and respiratory illnesses in the community in Bangladesh

1	Study ID number		
2	Name of child		
3	Sex of the child	1=Male<br>2=Female	
4	Date of birth (dd/mm/yyyy)		
5	Age of the child (in months)		
6	Name of the Village (code)		
7	Name of the Union (code)		
8	Name of the Upazila (code)		
9	Mobile number		
10	Follow up date (dd/mm/yyyy)		
10a	Follow up number		
11	Any Diarrhoea episodes in the last 14 days?	1=Yes<br>0=No; ***If No, skip to 22***	
12	Onset date of Diarrhea (dd/mm/yyyy)		
13	Onset time of Diarrhea, 24 hours (hh:mm)		
13a	Duration of diarrhea (days)		
14	Presence of visible blood in stool?	1=Yes<br>0=No	
15	If any other symptoms associated with Diarrhoea? (***Multiple response***)	1=Yes<br>0=No ***If No, skip to 16***	
	a) Fever	1=Yes<br>0=No	
	b) Abdominal pain	1=Yes<br>0=No	
	c) Vomiting	1=Yes<br>0=No	

	d) Cough and cold	1=Yes<br>0=No	☐
	e) Anorexia	1=Yes<br>0=No	☐
	f) Others, specify		
16	Where did you seek care for this episode of Diarrhoea? ***If Q16=4 to 10, skip to 17***	1=None<br>2=Self medication<br>3=Pharmacy<br>4=Private facility<br>5=UHC<br>6=FWC<br>7=Community clinic<br>8=District Hospital<br>9=Govt. Medical College Hospital<br>10=NGO Health worker<br>11=Others, specify	☐
16a	If not health facility, why (reason)?<br>***If Q16=(1,2,3,11)***	1=Long waiting time<br>2= High Transportation cost<br>3=Long distance<br>4=High Expense for treatment<br>5=Trust on local health care provider/pharmacy<br>6=Family problem<br>7=Others, specify	☐
16b	If Q16a=7 others, specify		
17	Did your child receive any antibiotics for Diarrhoea?	1=Yes<br>0=No; ***If No, skip to 18***	☐
17a	If Q17=1, Name of the antibiotic		
17b	If Q17=1, Dose of the antibiotic		
17c	If Q17=1, Source of antibiotic		
18	Did your child receive Zinc for Diarrhoea?	1=Yes<br>0=No	☐
19	Did your child receive Probiotic for Diarrhoea?	1=Yes<br>0=No	☐
20	Did your child receive Folic acid for Diarrhoea?	1=Yes<br>0=No	☐
21	Did your child receive ORS for Diarrhoea?	1=Yes; ***If Yes, skip to 22***<br>0=No	☐
21a	If Q21=0, reason of not taking ORS?	1=Did not know<br>2=Baby is too young to drink ORS<br>3=Baby is having cold and mother thinks ORS will aggravate cold	☐

		4=Wanted to consult the doctor first<br>5=Baby was reluctant to drink ORS<br>6=ORS was not available at home<br>7=Mother drank ORS instead, as the baby was breastfeeding<br>8=Visited pharmacy, but they did not give ORS<br>9=Others, specify	
21b	If Q21a=9 others, specify		
22	Anyone else from the family suffered from Diarrhoea in these two weeks?	1=Yes<br>0=No; ***If No, skip to 23***	☐
22a	If Q22=1, age of that patient (in months)		☐☐☐
22b	If Q22=1, Sex of that patient?	1=Male<br>2=Female	☐
22c	If Q22=1, Relation to the baby?	1=Mother, 2=Father, 3=Grandfather<br>4=Grandmother, 5=Sibling, 6=Cousins<br>7=Uncle/Aunty, 8=Other, specify	☐
22d	If Q22c=8 others, specify		
23	Any medication taken within the last 14 days for any illness other than diarrhoea?	1=Yes<br>0=No; ***If No, skip to 24***	☐
23a	If Q23=1, for which illness (specify)		
23b	If Q23=1, antibiotics taken in the past 14 days	1=Yes<br>0=No; ***If No, skip to 24***	☐
23c	If Q23b=1, Name of the antibiotic		
23d	If Q23b=1, Dose of the antibiotic		
23e	If Q23b=1, Source of the antibiotic		
24	Is the child currently breastfeeding	1=Yes<br>0=No	☐
25	Breast-fed for the first 6 months of life	1=Yes<br>0=No	☐
26	Exclusively breastfed for the first 6 months	1=Yes<br>0=No	☐
27	Remarks		
28	Field worker's initial	☐☐☐	
